# Cephalometric and occlusal changes of Class III malocclusion treated with or without extractions

**DOI:** 10.1590/2177-6709.25.4.024-032.oar

**Published:** 2020

**Authors:** Roberto Bombonatti, Arón Aliaga Del Castillo, Juliana Fraga Soares Bombonatti, Daniela Garib, Bryan Tompson, Guilherme Janson

**Affiliations:** 1 Universidade de São Paulo, Faculdade de Odontologia de Bauru, Departamento de Ortodontia, (Bauru/SP, Brazil).; 2 Universidade de São Paulo, Faculdade de Odontologia de Bauru, Departamento de Dentística, Endodontia e Materiais Odontológicos (Bauru/SP, Brazil).; 3 Hospital de Reabilitação de Anomalias Craniofaciais (Bauru/SP, Brazil).; 4 Faculty of Dentistry, University of Toronto, (Toronto/ON, Canada).; 5 Hospital for Sick Children, Division of Orthodontics (Toronto/ON, Canada).

**Keywords:** Cephalometric changes, Occlusal changes, Class III malocclusion, Orthodontic treatment

## Abstract

**Objective:**

The aim of this retrospective study was to evaluate the cephalometric and occlusal changes of orthodontically treated Class III malocclusion patients.

**Methods:**

The experimental groups comprised 37 Class III patients treated: G1) without (n=19) and G2) with extractions (n=18) . The control group (G3), matched by age and sex with the experimental groups, consisted of 18 subjects with untreated Class III malocclusion. Cephalometric (radiographs) and occlusal (study models) changes were assessed between the beginning (T_1_) and the end (T_2_) of treatment. Intergroup comparisons were performed with one-way ANOVA followed by Kruskal-Wallis tests (*p*< 0.05). Occlusal changes were evaluated by the peer assessment rating (PAR) index (ANOVA and Kruskal-Wallis tests), and the treatment outcomes were evaluated by the Objective Grading System (OGS) (*t*-tests).

**Results:**

The experimental groups showed a restrictive effect on mandibular anterior displacement and a discrete improvement in the maxillomandibular relationship. Extraction treatment resulted in a greater retrusive movement of the incisors and significant improvements in the overjet and molar relationship in both groups. The PAR indexes were significantly reduced with treatment, and the OGS scores were 25.6 (G1) and 28.6 (G2), with no significant intergroup difference.

**Conclusions:**

Orthodontic treatment of Class III malocclusion patients with fixed appliances improved the sagittal relationships, with greater incisor retrusion in the extraction group. Both the extraction and non-extraction treatments significantly decreased the initial malocclusion severity, with adequate and similar occlusal outcomes of treatment.

## RESUMO

**Objetivos:**

O objetivo desse estudo retrospectivo foi avaliar as alterações cefalométricas e oclusais de pacientes com má oclusão de Classe III tratados ortodonticamente.

**Método:**

Os grupos experimentais compreenderam 37 pacientes Classe III tratados: 19 com extrações dentárias (G1) e 18 sem (G2). O grupo controle (G3), compatibilizado em idade e sexo com os grupos experimentais, consistiu de 18 indivíduos com má oclusão de Classe III não tratada. Alterações cefalométricas (radiografias) e oclusais (modelos de estudo) foram avaliadas ao início (T_1_) e ao fim (T_2_) do tratamento. Comparações intergrupos foram realizadas com testes ANOVA a um critério e Kruskal-Wallis (*p*< 0,05). As alterações oclusais foram avaliadas pelo índice PAR (testes ANOVA e Kruskal-Wallis) e os resultados oclusais dos tratamentos, pelo índice *Objective Grading System* (OGS) (testes *t*).

**Resultados:**

Os grupos experimentais apresentaram um efeito restritivo no posicionamento anterior da mandíbula e uma discreta melhora na relação maxilomandibular. Os tratamentos com extrações resultaram em um maior movimento retrusivo dos incisivos e melhoras significativas no trespasse horizontal e na relação molar em ambos os grupos. Os índices PAR foram reduzidos significativamente com o tratamento, e os índices OGS foram iguais a 25,6 (G1) e 28,6 (G2), sem diferença significativa entre os grupos.

**Conclusões:**

O tratamento ortodôntico de pacientes com má oclusão de Classe III com aparelhos corretivos fixos melhorou as relações sagitais, com maior retrusão dos incisivos no grupo com extrações. Ambos os tratamentos, com e sem extrações dentárias, diminuíram significativamente a severidade inicial da má oclusão, com resultados oclusais do tratamento adequados e similares.

## INTRODUCTION

Class III malocclusion is a controversial subject among researchers concerning diagnosis, prognosis, and treatment, especially because of the unpredictable and potentially unfavorable nature of mandibular growth. The most common treatment alternatives for correction of this malocclusion include orthopedic devices in the mixed or early permanent dentition or in adolescent patients.^1,2^ In the permanent dentition, the treatment approaches may consist of fixed appliances associated with Class III elastics^3-5^ for dentoalveolar compensation, with or without extractions.^6-8^ Adult patients who present severe skeletal Class III deformity are usually potential candidates for orthognathic surgery to correct the skeletal anomaly.^9^

Studies described the dentofacial changes induced by orthopedic Class III treatment.^1,2^ However, except case reports, only a few have actually studied fixed appliances treatment changes in Class III malocclusion patients. The main effects of various approaches used in Class III malocclusion treatment are: maxillomandibular relationship and facial esthetics improvements, increase in lower anterior face height, protrusion of maxillary incisors, retroclination of mandibular incisors, correction of overbite, overjet and molar relationship.^3^ ^-^ ^6^ ^,^ ^8^ ^,^ ^10^ ^,^ ^11^ The treatment can be performed without extractions^10^ or with different extraction protocols.^11^ ^-^ ^14^

Few studies have compared the treatment effects with a control group to discriminate these changes from the usual craniofacial growth changes.^4,7,12^ Battagel and Orton^3^ used three groups: non-extraction treatment, mandibular premolar extraction treatment, and an untreated control group. In Class III treatment with fixed appliances, only Faerovig and Zachrisson^13^ and Janson et al^8^ assessed the occlusal changes on dental casts.

Therefore, the objective of this study was to compare the cephalometric and occlusal changes of Class III malocclusion patients treated with or without 4-premolar extractions and untreated Class III malocclusion subjects.

## MATERIAL AND METHODS

### Sample

This research was submitted and approved by the Ethics in Research Committee of Bauru Dental School, University of São Paulo (CAAE 48128915.6.0000.5417). Data was retrospectively and randomly obtained from files of two different study centres at the pre- (T_1_) and posttreatment (T_2_) (or observational) stages. Initially, all patients presented Class III molar relationship at least on one side. The sample size was calculated to be 17 patients (α=5%, β=20%, minimum difference = 2 mm and SD = 2 mm in Wits appraisal change).^4^

The experimental groups comprised patients treated at Bauru Dental School, University of São Paulo (Brazil). Group G1 consisted of 19 patients treated without extraction and group G2, of 18 patients treated with 4-premolar extractions protocol. Orthodontic treatment was performed with fixed Edgewise appliances, with 0.022 x 0.028-in conventional brackets and the usual archwire sequence (initial 0.014-in NiTi, followed by 0.016, 0.018, 0.020 and 0.019 x 0.025-in stainless steel archwires). According to the type of malocclusion, it was associated with rapid maxillary expansion using Hyrax appliance (due to transverse discrepancy) and Class III elastics, to correct the anteroposterior relationships. Crowding was corrected with expansion of the leveling archwires and stripping. In extraction treatment, the initial canine retraction was performed on a round continuous 0.014-in NiTi archwire and the anterior teeth retraction, with rectangular stainless steel archwires, both with elastomeric chains. The control group (G3) consisted of 18 subjects with untreated Class III malocclusion from the Burlington Growth Centre, located at Faculty of Dentistry, University of Toronto, Canada (Table 1).

**Table 1:** Intergroup comparability.

	G1		G2		G3		p
	Mean	(SD)	Mean	(SD)	Mean	(SD)	
T_1_ (age in years)	14.3	(2.5)	14.8	(2.3)	14.0	(1.2)	0.571 §
T_2_ (age in years)	17.6	(2.5)	17.7	(2.4)	17.8	(2.3)	0.972 §
Treatment or observational time (years)	3.3	(1.4)	3.0	(0.8)	3.8	(2.4)	0.974 €
SEX							
Male	7 (12.7%)		7 (12.7%)		10 (18.2%)		0.458 Ω
Female	12 (21.8%)		11 (20.0%)		8 (14.6%)		

§ = ANOVA test. € = Kruskal-Wallis test. Ω = Chi-square test.

### Cephalometric evaluation

The lateral cephalometric radiographs were hand traced by one examiner. The image magnification factors of the radiographs ranged from 6% to 9.8%. The cephalometric tracings were then digitized (Lexmark, model MX810 Series, Lexington, Kentucky, USA) and analyzed with Dolphin Imaging v. 11.7 Premium software (Patterson Dental Supply, Inc., Chatsworth, California, USA). Twenty seven variables were used: 11 angular and 16 linear (Table 2).

**Table 2:** Cephalometric variables.

VARIABLES	
MAXILLARY COMPONENT	
SNA (degrees)	SN to NA angle
A-NPerp (mm)	Linear distance from A-point to nasion-perpendicular
Co-A (mm)	Condylion to A-point distance (effective maxillary length)
MANDIBULAR COMPONENT	
SNB (degrees)	SN to NB angle
P-NPerp (mm)	Linear distance from pogonion to nasion-perpendicular
Co-Gn (mm)	Condylion to gnathion distance (effective mandibular length)
MAXILLOMANDIBULAR RELATIONSHIP	
ANB (degrees)	NA to NB angle
Wits (mm)	Distance between perpendicular projections of points A and B on the functional occlusal plane
NAP (degrees)	Angle between points N, A, and P
GROWTH PATTERN	
SN.GoGn (degrees)	SN to GoGn angle
ANS-Me (mm)	Distance from ANS to menton (lower anterior face height)
SN.OccPlane (degrees)	SN to occlusal plane angle
MAXILLARY DENTOALVEOLAR COMPONENT	
1.NA (degrees)	Maxillary incisor long axis to NA angle
1-NA (mm)	Distance between most anterior point of crown of maxillary incisor and NA line
1-PP (mm)	Perpendicular distance between incisal edge of maxillary incisor and palatal plane
1.PP (degrees)	Maxillary incisor long axis to palatal plane angle
1-AP (mm)	Distance between most anterior point of crown of maxillary incisor and A-P line
MANDIBULAR DENTOALVEOLAR COMPONENT	
1.NB (degrees)	Mandibular incisor long axis to NB angle
1-NB (mm)	Distance between most anterior point of crown of mandibular incisor and NB line
1-PM (mm)	Perpendicular distance between incisal edge of mandibular incisor and mandibular plane
1.PM (degrees)	Mandibular incisor long axis to mandibular plane (Go-Me) angle
DENTAL RELATIONSHIPS	
Overbite (mm)	Distance between the incisal edges of maxillary and mandibular incisors, perpendicular to the occlusal plane
Overjet (mm)	Distance between the incisal edges of maxillary and mandibular incisors, parallel to the occlusal plane
Molar Relation (mm)	Distance between mesial points of maxillary and mandibular molars, parallel to Frankfort plane
SOFT TISSUE	
UL-S Line (mm)	Perpendicular distance between line S and UL (most anterior point of upper lip)
LL-S Line (mm)	Perpendicular distance between line S and LL (most anterior point of lower lip)
G’.Sn.P’ (degrees)	G’Sn to SnP’ angle (Facial Convexity)

### Occlusal evaluation

The occlusal changes were measured by the same examiner on dental casts using the Peer Assessment Rating (PAR) index^15^ with a 0.01-mm precision digital caliper (Mitutoyo Corp, Kanogawa, Japan). Higher scores indicate higher levels of irregularity. This evaluation quantified the initial malocclusion severity (PAR_1_), the occlusal treatment results (PAR_2_), the PAR treatment or observation changes (PAR_2-1_) and the percentage of PAR change (%PAR), using the following formula^16^:

$$\%PAR=\;\left(PAR_{2-1}/PAR_{1}\right)\times 100$$

The quality of the occlusal and radiographic results of the orthodontic treatments was evaluated with the Objective Grading System (OGS), recommended by The American Board of Orthodontics.^17^ For each parameter that deviates from ideal, points are subtracted according to the problem severity. An ideal occlusion and alignment achieve a score of 0 points.

#### Error study

After 28 days, the same examiner remeasured 12 radiographs and 12 dental casts randomly selected, to calculate the random errors with Dahlberg’s formula^18^ and the systematic errors with dependent *t*-tests (*p*< 0.05).^19^

#### Statistical analysis

Kolmogorov-Smirnov tests were performed to check for normal distribution. Intergroup age comparability was evaluated with one-way ANOVA (normal distribution) and Kruskal-Wallis (non-normal distribution) tests. Chi-square test was used to evaluate intergroup sex distribution.

Analysis of variance, followed by Tukey tests, was performed to compare the cephalometric and occlusal statuses at T_1_ and the treatment or observation changes (T_2_-T_1_) of the groups. Variables without normal distribution were compared with Kruskal-Wallis, followed by Duncan tests. Intergroup comparison of the OGS was performed with *t*-test. All statistical analyses were performed with Statistica software (v. 7.0; StatSoft Inc., Tulsa, Okla, USA) at a significance level of *p*< 0.05.

### RESULTS

There were no significant systematic errors and the cephalometric random errors ranged from 0.27mm (overjet) to 2.25mm (Pg-NPerp). The occlusal random errors ranged from 0.91 (PAR) to 1.40 (OGS). The groups were comparable regarding initial and final age, treatment or observational time, and sex distribution (Table 1).

Group 1 showed greater mandibular protrusion and length, which contributed to the more accentuated skeletal Class III relationship and a significantly greater profile concavity than the other groups (Table 3). The growth pattern of this group was also slightly more horizontal. The treatment groups showed greater labial tipping and significantly greater protrusion of the maxillary incisor and Class III molar relationship severity than the control group. The soft tissue characteristics were similar between the groups.

**Table 3:** Intergroup comparison before treatment (T1, ANOVA, followed by Tukey tests).

	G1		G2		G3		P
	Mean	(SD)	Mean	(SD)	Mean	(SD)	
MAXILLARY COMPONENT							
SNA	83.9	4.2	81.6	4.7	83.7	3.0	0.169
A-NPerp	-0.7	3.7	-0.3	4.0	-1.6	3.2	0.520
Co-A	83.0	3.9	82.1	3.7	81.4	2.9	0.378
MANDIBULAR COMPONENT							
SNB	85.5^A^	4.0	81.8^B^	4.5	82.9^AB^	2.5	0.012*
P-NPerp	4.0^A^	5.3	1.1^AB^	6.8	-2.6^B^	6.7	0.009*
Co-Gn	116.9^A^	6.8	112.7^AB^	5.6	109.8^B^	4.4	0.002*
MAXILLOMANDIBULAR RELATIONSHIP							
ANB	-1.6^A^	1.8	-0.2^AB^	1.9	0.8^B^	1.8	0.001*
Wits	-5.9	2.4	-4.6	2.6	-4.2	2.3	0.086
NAP	-6.0^A^	4.2	-1.8^B^	4.6	-0.6^B^	4.6	0.001*
GROWTH PATTERN							
SN.GoGn	28.6^A^	4.3	33.5^B^	4.5	30.6^AB^	4.4	0.005*
ANS-Me	65.2^A^	5.0	64.5^AB^	3.9	61.6^B^	3.6	0.033*
SN.OccPlane	11.1	5.0	14.3	5.3	14.5	3.4	0.055
MAXILLARY DENTOALVEOLAR COMPONENT							
1.NA	31.7^A^	6.2	30.2^AB^	5.5	26.0^B^	4.8	0.008*
1-NA	6.4^A^	2.1	6.8^A^	2.5	4.7^B^	1.4	0.007*
1-PP	26.8	2.1	26.9	2.8	25.7	1.9	0.209
1.PP	120.0	5.2	119.1	6.0	117.2	5.5	0.294
1-AP	4.3	2.3	6.1	2.5	4.4	2.3	0.051
MANDIBULAR DENTOALVEOLAR COMPONENT							
1.NB	23.7	6.3	26.4	6.4	23.1	7.9	0.308
1-NB	3.1	2.1	4.9	2.7	3.7	2.4	0.088
1-MP	37.3	3.1	37.4	2.1	35.5	2.9	0.071
1.MP	86.9	7.4	88.6	6.6	86.9	9.4	0.760
DENTAL RELATIONSHIPS							
Overbite	0.5	1.2	0.4	1.1	0.8	0.7	0.453
Overjet	1.3	1.7	1.7	1.7	2.1	0.9	0.279
Molar Relation	-4.6^A^	1.4	-4.5^A^	1.7	-3.3^B^	1.4	0.022*
SOFT TISSUE							
UL-S Line	-2.2	2.2	-0.7	2.5	-0.5	2.4	0.073
LL-S Line	-0.2	2.7	2.0	2.6	0.6	3.5	0.081
G’.Sn.P’	172.5	5.3	168.4	5.1	170.0	4.7	0.079

* Statistically significant at p < 0.05. Different superscript letters represent statistically significant differences.

The orthodontic treatment improved the maxillomandibular relationship due mainly to the tendency of a more restrictive effect on mandibular anterior displacement, when compared to the control group (Table 4).Group 1 showed significantly greater advancement of the maxilla than the other groups. Groups G1 and G3 showed slight increase in maxillary incisor protrusion, whereas group G2 showed retrusion. In general, the mandibular incisors had greater retrusion and vertical control in G2 than in the other groups. The treated groups showed significantly greater improvement in molar relationship and in the overjet. The soft tissue changes were similar in the groups.

**Table 4:** Intergroup comparison of treatment and growth changes (T2-T1, ANOVA, followed by Tukey tests).

	G1		G2		G3		P
	Mean	(SD)	Mean	(SD)	Mean	(SD)	
MAXILLARY COMPONENT							
SNA	0.3	2.0	0.4	1.4	0.4	2.5	0.990
A-NPerp	0.6^A^	2.2	-1.5^B^	2.7	0.3^AB^	2.3	0.023*
Co-A	0.5^A^	2.4	0.3^A^	1.8	2.7^B^	3.3	0.011
MANDIBULAR COMPONENT							
SNB	-0.1	1.6	0.5	1.6	1.4	2.4	0.077
P-NPerp	1.4^AB^	4.1	-1.9^A^	5.0	2.3^B^	5.1	0.027*
Co-Gn	2.5	3.8	2.2	2.9	6.8	6.8	0.154 €
MAXILLOMANDIBULAR RELATIONSHIP							
ANB	0.4^A^	1.4	-0.1^AB^	1.1	-1.0^B^	1.5	0.012*
Wits	1.6^A^	2.2	0.9^A^	2.5	-0.9^B^	1.3	0.001*
NAP	-0.1	2.9	-1.0	2.4	-1.9	3.3	0.175
GROWTH PATTERN							
SN.GoGn	0.1	2.2	-0.3	2.3	-0.5	2.8	0.780
ANS-Me	2.1	2.8	2.3	3.0	4.1	4.2	0.171
SN.OccPlane	-1.9	2.7	-1.8	3.6	-1.6	3.0	0.956
MAXILLARY DENTOALVEOLAR COMPONENT							
1.NA	2.5	6.6	-1.5	5.8	1.6	2.8	0.064
1-NA	1.0^A^	2.3	-1.0^B^	2.0	0.8^A^	1.2	0.004*
1-PP	0.4	1.2	0.8	1.2	1.1	2.0	0.312
1.PP	2.8	6.7	-1.3	5.7	2.1	2.9	0.058
1-AP	0.9^A^	1.8	-1.4^B^	1.6	0.1^A^	0.9	0.000*
MANDIBULAR DENTOALVEOLAR COMPONENT							
1.NB	-1.6^A^	4.5	-5.1^B^	4.1	-2.1^AB^	3.8	0.025*
1-NB	0.3^A^	1.3	-2.1^B^	1.4	-0.1^A^	1.3	0.000*
1-MP	1.7^AB^	2.2	0.6^A^	1.8	2.4^B^	2.1	0.032*
1.MP	-1.5^A^	4.3	-5.3^B^	4.0	-3.1^AB^	3.8	0.023*
DENTAL RELATIONSHIPS							
Overbite	0.4	1.4	0.6	1.4	-0.2	0.7	0.153
Overjet	1.1^A^	1.8	1.0^A^	1.5	-0.4^B^	0.7	0.003*
Molar Rel	1.0^A^	1.2	1.6^A^	1.8	0.1^B^	2.3	0.047*
SOFT TISSUE							
UL-S Line	-0.1	1.4	-0.4	1.1	0.1	2.7	0.716
LL-S Line	-0.1	1.2	-1.2	0.8	0.4	3.4	0.078
G’.Sn.P’	-0.3	3.1	0.2	2.3	2.5	5.4	0.079

* Statistically significant at p<0.05. Different superscript letters represent statistically significant differences. € = Kruskal-Wallis test.

Initially, group G2 presented significantly greater occlusal Class III malocclusion severity (Table 5). Malocclusion severity of groups G1 and G2 clearly was significantly reduced with treatment, whereas in the untreated patients it increased. Although the malocclusion reduction amount was significantly greater in G2, the percentage of PAR improvement was similar in the treated groups, as well as the final quality of orthodontic treatment.

**Table 5:** Intergroup comparison of OGS and PAR indexes.

	G1		G2		G3		P
	Mean	(SD)	Mean	(SD)	Mean	(SD)	
PAR1	28.1^A^	11.2	36.2^B^	8.1	21.1^A^	10.8	0.000 §*
PAR2	3.7^A^	4.5	5.2^A^	2.7	25.0^B^	9.7	0.000 €*
PAR2-1	-24.4^A^	10.3	-30.9^B^	7.1	3.9^C^	6.5	0.000 §*
%PAR	-87.6^A^	13.2	-85.7^A^	5.8	39.2^B^	71.6	0.000 €*
OGS	25.6	9.2	28.6	6.0	---	---	0.085 ¥

* Statistically significant at p<0.05. Different superscript letters mean statistically significant differences.

§ = ANOVA followed by Tukey tests. € = Kruskal-Wallis followed by Duncan tests. ¥ = t test.

## DISCUSSION

### Sample characteristics

Despite the limitation of a retrospective study, the group characteristics were similar, especially considering the difficulty in obtaining an untreated Class III malocclusion control group (Tables 1 and 3). Although there were significant differences regarding some cephalometric variables at baseline between control and treatment groups, one has to bear in mind that the changes in groups with similar malocclusions are the most important issues to be evaluated in this type of investigation. Few studies used a control group of untreated Class III patients.^3^ ^,^ ^4^ ^,^ ^7^ ^,^ ^12^

### Cephalometric changes

The orthodontic treatments provided smaller mandibular advancement with respect to the control, especially in the extraction group, improving the maxillomandibular relationship (Table 4). Less protrusion or greater mandibular retrusion is evidenced in compensatory Class III orthodontic treatment in growing patients with orthopedic appliances, aiming at altering the skeletal growth pattern of the patients and either advance the maxilla forward or prevent the further forward growth of the mandible through a clockwise rotation, or both.^3,4,7^ This effect is less evident in adult patients, where the maxillary dentoalveolar compensation is greater.^6^ ^,^ ^8^ In the current study, the maxilla does not appear to have undergone major changes in relation to its initial positioning,^3^ possibly due to the initial mean age of the sample. In order to obtain more maxillary skeletal effects, treatment must be instituted before the pubertal growth spurt.^1^ The extraction group demonstrated greater maxillary anterior displacement restriction than the non-extraction group, probably due to the greater maxillary incisor retraction in order to close the extraction spaces.^20^ ^,^ ^21^ Thus, it may be stated that minimal skeletal (maxillomandibular) changes were observed, while the dentoalveolar effects were more pronounced.

An increase in lower anterior face height (LAFH, ANS-Me) during Class III malocclusion treatment may result in a more retrusive position of the mandible, improving the sagittal relationship.^4^ ^,^ ^8^ Although the LAFH increased with treatment and growth, no intergroup difference was observed, similarly to previous investigations.^7,12^ Both treatment protocols did not cause significantly different changes in the growth pattern, compared with untreated subjects. Counterclockwise rotation of the occlusal plane has been observed in treatment with Class III elastics.^11,14^ However, this was not observed in the treated groups.

Only the extraction group showed significantly greater maxillary incisor retraction than the other groups (Table 4). This was probably due to retraction of these teeth in order to close the extraction spaces. In Class III non-extraction mechanics in the maxillary arch, the incisors experience only small changes.^4^ ^,^ ^11^ ^,^ ^12^ ^,^ ^14^ ^,^ ^22^ There are no studies specifically evaluating the extraction effects on the maxillary incisors in Class III treatment with fixed appliances.

The extraction group presented significantly greater mandibular incisor lingual tipping and retrusion, primarily in relation to the non-extraction group. In relation to the control group, it showed only significant retrusion (Table 4). Other studies with mandibular extractions observed incisor lingual tipping and retrusion^3^ or only lingual tipping,^12^ in relation to the control groups. There were significant improvements of the overjet and molar relationship in both treated groups, in relation to the control. It was fortunate that, despite the small changes in maxillary and mandibular incisors in the non-extraction group, significant improvements of these variables were possible. Another study showed similar changes in overjet and molar relation, without significant incisor changes.^4^

Even though these are compensatory treatments where facial impact is not as evident as in orthodontic-surgical treatment, changes of soft tissue may camouflage the skeletal Class III discrepancy, such as reduction of lower lip projection due to mandibular incisor retrusion^11^ ^,^ ^14^ and the increase in facial convexity.^3,7,11,14^ In the current study, although there were soft tissue changes, they were very discrete and statistically similar to the growth changes.

### Occlusal changes

The extraction group had greater initial malocclusion severity, resulting from greater crowding (displacement), which weighted toward an extraction approach in this group (Table 5). The orthodontic treatments significantly improved the occlusion, when compared to the untreated group, which even had an increase in malocclusion severity, overtime. The final PAR indexes in the treated groups were similar between the groups and also similar to previous reports on treated Class III malocclusion cases.^22^ Because the initial malocclusion severity was significantly greater in G2, the amount of PAR reduction (PAR_2-1_) was also significantly greater than in G1. However, the percentage of PAR reduction was similar in the treated groups G1 and G2 (%PAR). Orthodontic treatment is considered adequate when the index reduction is greater than 70%,^23^ and therefore, treatment provided in the groups showed adequate quality.

The similar OGS in the treated groups confirm the results of the PAR_2_, showing that both groups had similar quality of finishing (Table 5). No previous studies have compared the quality of occlusal finishing in Class III malocclusion treatments with or without extractions.

The American Board of Orthodontics states a case report with scores above 30 points will fail.^17,24^ Some investigations in different universities showed mean OGS scores of 22.1^25^, 22.4^26^, 32.2^27^ and 33.6,^28^ comparable with the present results. In addition, the fact that the patients were treated in an university does not seem to influence the results, since studies report no differences between orthodontic treatment outcomes in university clinics and private practices.^29^ ^,^ ^30^

## CONCLUSIONS

The effects of non-extraction and extraction Class III malocclusion treatments were:

» Orthodontic treatment with fixed appliances improved the sagittal relationships mainly with dentoalveolar changes.
» Extraction treatment presented greater maxillary and mandibular incisor retrusion, when compared to non-extraction treatment.
» Both extraction and non-extraction treatments significantly decreased the initial malocclusion severity in the same proportion.
» The final quality of orthodontic treatment was statistically similar between the extraction and non-extraction groups, with adequate occlusal outcomes.
