# ERRATUM

**DOI:** 10.1590/2177-6709.25.6.err.002

**Published:** 2020

The original article *“Cephalometric and occlusal changes of Class III malocclusion treated with or without extractions”*, with DOI: 10.1590/2177-6709.25.4.024-032.oar, published in Dental Press J. Orthod. vol.25 no.4 Maringá July/Aug. 2020 Epub Sep 21, 2020, presented the following authors: 

Roberto Bombonatti, **Arón** Aliaga Del Castillo, Juliana Fraga Soares Bombonatti, Daniela Garib, Bryan Tompson and Guilherme Janson

The “How to cite this article” presented the following information (**currently in PubMed**): 

Bombonatti R, **Castillo AAD**, Bombonatti JFS, Garib D, Tompson B, Janson G. Cephalometric and occlusal changes of Class III malocclusion treated with or without extractions. Dental Press J Orthod. 2020 Jul-Aug;25(4):24-32. doi: 10.1590/2177-6709.25.4.024-032.oar. PMID: 32965384.

Now the article should have the following row of authors and “How to cite this article”: 

Roberto Bombonatti, **Aron** Aliaga-Del Castillo, Juliana Fraga Soares Bombonatti, Daniela Garib, Bryan Tompson and Guilherme Janson

Bombonatti R, **Aliaga-Del Castillo A**, Bombonatti JFS, Garib D, Tompson B, Janson G. Cephalometric and occlusal changes of Class III malocclusion treated with or without extractions. Dental Press J Orthod. 2020 Jul-Aug;25(4):24-32. doi: 10.1590/2177-6709.25.4.024-032.err. PMID: 32965384.

